# *Staphylococcus epidermidis* Endophthalmitis Masquerading as Panuveitis After an Imperceptible Ocular Trauma

**DOI:** 10.4274/tjo.galenos.2018.44520

**Published:** 2019-02-28

**Authors:** Juan Martin Sanchez, Diego Almeida, Tareq Jaouni, Radgonde Amer

**Affiliations:** 1 Hadassah University Hospital, Ophthalmology Clinic, Jerusalem, Israel

**Keywords:** Diagnostic vitrectomy, exogenous endophthalmitis, staphylococcus epidermidis, ocular trauma, penetration trauma

## Abstract

Endophthalmitis after a penetrating trauma occurs in 3% to 30% of cases. Prompt recognition and treatment are paramount to avoid irreversible visual loss. We present a case of severe panuveitis following ocular trauma with a tree branch that did not cause any evident ocular wound and discuss the difficulties in achieving a diagnosis that can allow proper treatment. A healthy 21-year-old man presented with acute anterior uveitis. He was managed elsewhere with oral acyclovir and topical steroids for presumed herpetic uveitis. He subsequently developed severe panuveitis with profound decrease in vision. Diagnostic vitrectomy was performed and vitreous samples were positive for *Staphylococcus epidermidis*. Systemic and intravitreal antibiotic therapy was initiated and after 5 days, the patient recovered with a remarkable improvement in visual acuity to 6/12. Post-traumatic endophthalmitis can result from an imperceptible trauma with no obvious compromise of the globe.

## Introduction

Endophthalmitis is a microorganismal infection caused exclusively by bacteria or fungi within the structures of the eye, including the aqueous and vitreous humor. Most cases are caused by microbial inoculation from an exogenous source, usually after blunt or penetrating trauma, surgery, foreign body, or as a complication of an eye infection such as keratitis or conjunctivitis. Bacterial infection is the most common type and the clinical presentation is typically acute. Acute bacterial endophthalmitis is a serious sight-threatening condition that must be addressed as an emergency. Endophthalmitis consequent to penetrating eye trauma has been reported in 3% to 30% of the cases.^1,2^

We present the case of a healthy young male who developed serious acute *Staphylococcus epidermidis *endophthalmitis following an imperceptible trauma that did not overtly compromise the integrity of the eye globe.

## Case Report

A healthy 21-year-old man was admitted to our hospital after being referred from another medical institution because of worsening left eye (LE) uveitis. He was treated 3 weeks earlier with oral acyclovir and topical steroids because of suspected LE herpetic anterior uveitis as serological tests revealed positive immunoglobulin M to herpes simplex virus-1. Initially he responded to treatment but 2 weeks later, his vision declined from 6/9 to counting fingers (CF), resulting in his referral to our center. 

The patient denied any relevant past medical history. However, he mentioned that a month earlier he was examined for LE discomfort and diagnosed with allergic conjunctivitis following minor blunt trauma from a tree branch. 

**Figure 1:**
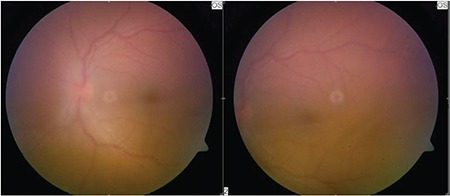
Color fundus photograph of the left eye showing hazy fundus view because of dense vitritis

On examination, visual acuity (VA) was 6/6 in the right eye (RE) and CF at 1 meter in the LE. Intraocular pressure was 14 mmHg in both eyes. RE anterior and posterior segments were normal. There was a remarkable LE anterior chamber reaction with dust-like keratic precipitates, cells (4+), flare (2+), and some iris nodules. Fundus assessment was not possible because of dense vitritis. B-scan and high-frequency ultrasound did not reveal any intraocular foreign body. Aqueous tap was performed and the sample was sent for culture and polymerase chain reaction analysis. It was negative for all herpes viruses and for 16S rDNA. Meanwhile, with oral steroids and valacyclovir, the patient showed signs of improvement and LE VA improved to 6/15. Despite the remarkable improvement, it was insufficient as the patient continued to have marked anterior uveitis and vitritis. A white shadow was noted in the peripheral temporal retina of the LE which again could not be assessed properly due to vitreous opacities (Figure 1). The patient eventually underwent pars plana vitrectomy and laser retinopexy was performed around the white peripheral temporal lesion, which was later believed to be the site of penetrating injury by a thorn on the tree branch from the previous trauma described by the patient. Gram staining of the undiluted vitreous samples showed gram-positive cocci. 16S rDNA was positive for *S. epidermidis*, and blood agar and chocolate agar cultures confirmed the result with moderate growth. 

The patient was treated with intravitreal antibiotics (vancomycin 1 mg/0.1 mL and ceftazidime 2.25 mg/0.1 mL) and intravitreal dexamethasone 400 mcg/0.1 mL as well as intravenous (IV) vancomycin (1 g twice/day), and oral prednisone was continued. 

After 48 hours of treatment, the patient showed remarkable clinical improvement. LE VA was 6/12. There was no need to administer more intravitreal antibiotics. 

After 5 days of IV antibiotic treatment, the patient was discharged on topical and a tapering regimen of oral steroids.

**Figure 2:**
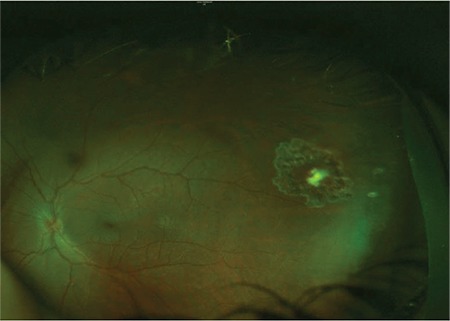
Color fundus photograph of the left eye 2 weeks postoperatively showing a clear fundus view (binocular indirect ophthalmoscopy score of zero) with normallooking optic disc, macula, and retinal vessels. However, a white scar surrounded by laser marks is visible in the temporal peripheral retina, indicating the site of the penetrating injury

After a follow-up period of 3 months, LE VA was 6/6 with complete resolution of the infectious process (Figure 2).

## Discussion

The normal bacterial flora of the human body is composed of gram-positive and gram-negative microorganisms, primarily *S. epidermidis* and *Propionibacterium acnes*, which are isolated under normal conditions from the skin, eyelid margins, conjunctival sac, and mucosal tissues.^3^

*S. epidermidis* is the most representative of this bacterial flora since it is commonly found in ocular surface isolates.^4^In our patient’s case, this microorganism was able to initiate and perpetuate the inflammatory response via a traumatic mechanism that allowed it to colonize the vitreous cavity and retina. The peripheral retinal temporal scar appeared to be the site of injury from a fine thorn that inoculated the bacteria, subsequently leading to severe endophthalmitis. 

Sabaci et al.^2^ reported in a retrospective study of 228 eyes with deadly-weapon-related open-globe injuries that *S. epidermidis* was the most common isolate and that infection with this less virulent microbe was the only factor associated with favorable outcome in their series. Our patient sustained a minor, imperceptible trauma without any evidence of penetrating intraocular foreign body on biomicroscopic examination, thus complicating the clinical picture. 

Risk factors for the development of post-traumatic endophthalmitis have been examined by several groups. Essex et al.^5^ analyzed the clinical course and visual outcomes of 250 consecutive patients admitted to a single ophthalmic hospital with open-globe injuries. The following factors were associated with the subsequent development of endophthalmitis: dirty wound, retained intraocular foreign body (IOFB), lens capsule breach, delayed primary repair, and residing in a rural area. 

In the case of our patient, the injury likely occurred in a rural setting, but there was no lens capsule rupture or delayed repair of the inciting wound because it was most probably a self-sealing wound that did not lead to clinically visible signs. 

The use of systemic and intraocular antibiotics for prophylaxis against post-traumatic endophthalmitis remains controversial. Nevertheless, when systemic antibiotics are not employed after open-globe injuries, there is a greater risk for endophthalmitis development.^6^ In a prospective, randomized study, cases of IOFB that were managed with intracameral and intravitreal antibiotics were associated with a reduced risk of endophthalmitis compared with the control group treated with intravitreal balanced salt solution.^2^

In conclusion, this case illustrates that post-traumatic endophthalmitis can occur with an imperceptible trauma without obvious compromise of the globe. Thorough clinical history is needed in order to properly assess subtle signs and ensure a satisfactory outcome.
